# IL-6-driven metabolic reprogramming of the multiple myeloma microenvironment

**DOI:** 10.1186/s40170-026-00451-4

**Published:** 2026-08-06

**Authors:** María Matamala Montoya, Silke Eleveld, Esther A. Zaal, Celia R. Berkers

**Affiliations:** 1 Division Cell Biology, Metabolism & Cancer, Department Biomolecular Health Sciences, Faculty of Veterinary Medicine, Utrecht University, Utrecht, The Netherlands; 2 Biomolecular Mass Spectrometry and Proteomics, Bijvoet Center for Biomolecular Research and Utrecht Institute of Pharmaceutical Sciences, Utrecht University, Utrecht, Netherlands

**Keywords:** Multiple myeloma, Bortezomib, Cancer metabolism, TME, IL-6, Metabolomics, Metabolism

## Abstract

**Background:**

Multiple myeloma is an aggressive plasma cell malignancy that remains incurable despite major therapeutic advances and depends on extensive metabolic remodeling to support malignant growth and drug resistance. Although tumor cell-intrinsic mechanisms of drug resistance are well characterized, the contribution of cells in the tumor microenvironment, particularly bone marrow stromal cells, to metabolic remodeling and therapeutic resistance remains poorly understood.

**Methods:**

We combined [U-^13^C]glucose tracing metabolomics with a metabolomics-compatible co-culture system of multiple myeloma and bone marrow stromal cells to assess the impact of co-culture on metabolic pathway activity across genetically distinct multiple myeloma cell lines. Cells were additionally subjected to cytokine analysis, gene expression analysis and drug response assays to investigate associations between metabolic changes, treatment responses and cytokine abundances.

**Results:**

Bone marrow stromal cell co-culture induced a convergent metabolic rewiring in multiple myeloma cells, enhancing pathways previously linked to bortezomib resistance, including glycolysis, tricarboxylic acid cycle activity, serine synthesis and nucleotide biosynthesis. In parallel, stromal cells acquired a cancer-associated fibroblast-like phenotype characterized by increased interleukin-6 and transforming growth factor-β production. This cytokine milieu promoted bortezomib resistance and stromal cell activation. Interleukin-6 alone was sufficient to rescue multiple myeloma cells from bortezomib-induced cytotoxicity and reproduced a substantial fraction of the co-culture-induced metabolic remodeling. Analysis of patient proteomics revealed a strong positive association between interleukin-6 abundance and expression of enzymes involved in mitochondrial metabolism, glycolysis, serine metabolism, nucleotide metabolism and redox homeostasis.

**Conclusions:**

These findings identify bone marrow stromal cell-derived interleukin-6 as a central driver of tumor microenvironment-mediated metabolic reprogramming and bortezomib resistance in multiple myeloma. Targeting interleukin-6-dependent metabolic signaling may represent a promising strategy to overcome stromal-driven therapeutic resistance.

**Clinical trial number:**

Not applicable.

**Supplementary Information:**

The online version contains supplementary material available at https://doi.org/10.1186/s40170-026-00451-4.

## Introduction

Multiple myeloma (MM) is an incurable B-cell neoplasm that develops in the bone marrow [1]. Like many cancers, MM depends on extensive metabolic remodeling to support malignant growth [2, 3]. MM cells exhibit elevated glycolysis (Warburg effect) [4, 5], channeling glucose-derived carbon into anabolic pathways to produce energy, building blocks for macromolecules and sustain redox balance needed for proliferation [6].

Resistance to therapy, particularly to the proteasome inhibitor bortezomib (BTZ), is associated with additional metabolic rewiring [7–12]. BTZ-resistant MM cells show increased glycolysis with enhanced glucose-derived carbon incorporation into the pentose phosphate pathway (PPP) and serine synthesis pathway (SSP) through increased expression of 3-phosphoglycerate dehydrogenase (PHGDH). These adaptations support glutathione production and redox homeostasis, thereby protecting MM cells from BTZ-induced cytotoxicity [9]. In addition, BTZ-resistant cells have higher mitochondrial activity, characterized by enhanced glutamine-driven tricarboxylic acid (TCA) cycle activity and oxidative phosphorylation (OXPHOS) [8]. This metabolic rewiring results in dependencies on specific pathways and metabolic vulnerabilities that offer new opportunities for therapeutic intervention [8].

The tumor microenvironment (TME) plays a central role in shaping these metabolic adaptations. Within the MM bone marrow niche, bone marrow stromal cells (BMSCs), immune cells, endothelial cells and extracellular matrix components collectively influence nutrient availability, exchange metabolites and activate pro-survival signaling pathways [6, 13–16]. Among these, BMSCs are particularly influential, supporting MM cell proliferation, survival and metabolism [17–19]. In MM patients, BMSCs frequently acquire a pro-tumorigenic cancer-associated fibroblast (CAF)-like phenotype, driven by microenvironmental cues, such as transforming growth factor-β (TGF-β) [20–22]. This CAF-like state is marked by elevated CAF markers such as α-smooth muscle actin (α-SMA) and fibroblast activation protein (FAP) [22–25], which are increased in relapsed MM patients and linked to drug resistance [22]. CAFs promote drug resistance through direct adhesion-mediated interactions and the release of soluble factors, including interleukin-6 (IL-6), tumor necrosis factor alpha (TNF-α) and metabolites [26–31]. Although the MM TME drives metabolic reprogramming, the specific metabolic alterations and cytokine-driven mechanisms that initiate and sustain these changes remain largely unknown.

We previously showed that co-culture of RPMI-8226 MM cells with BMSCs increases activity of metabolic pathways linked to BTZ, such as glycolysis, mitochondrial activity and antioxidant defenses [6]. However, RPMI-8226 represents a MM subtype that is infrequent in patients [32, 33]. Here, we incorporate a heterogeneous panel of MM cell lines in a metabolomics-compatible MM-BMSC co-culture model to dissect how the TME shapes metabolic remodeling and to identify key drivers of this process. By integrating cytokine profiling with isotope-labeling strategies, we characterize microenvironment-driven metabolic remodeling and identify IL-6 as a critical mediator, linking stromal signaling to metabolic adaptation and drug resistance. Together, these findings uncover an IL-6-metabolism axis that underpins therapeutic resistance in MM and provide a mechanistic basis for metabolically targeted interventions.

## Materials and methods

### Reagents

Bortezomib (BTZ) was purchased from Selleck Chemicals. Recombinant Human IL-6 (carrier-free) was purchased from Biolegend Europe B.V. and diluted in sterile 0.1% BSA in PBS. TGF-β1 mouse recombinant was purchased from eBioscience and diluted in 0.1% BSA in PBS. In all experimental conditions, the control contained the same solvent concentration of the drug. All solvents were obtained from Biosolve and all other chemicals were obtained from Sigma-Aldrich, unless stated otherwise.

### Cell lines

Human HS-5 and RPMI-8226 cells were a kind gift from Richard Groen and Gerrit Jansen (Amsterdam University Medical Center, The Netherlands). Human MM AMO1 cells were kindly provided by C. Driessen (Kantonsspital St. Gallen, Switzerland). Human MM U266 cells were kindly provided by Monique Minnema (University Medical Centre Utrecht, The Netherlands). All cell lines were tested for mycoplasma contamination every two months.

Cells were cultured in RPMI-1640 medium (Gibco, #21870076), supplemented with 2 mM L-glutamine (Gibco, #25030081), 10% fetal bovine serum (FBS, Gibco, #16000044) and 100 μg/mL penicillin/streptomycin (Gibco, #15140122). Cells were kept at 37 °C in humidified 5% CO_2_ atmosphere. For all experiments, MM cells were diluted in fresh medium 1 day in advance. Experiments were performed in RPMI-1640 medium unless otherwise stated.

### Co-culture assays

For co-culture (CC) experiments, 3.8 × 10^5^ HS-5 cells (hereafter referred to as BMSCs) were seeded at the bottom of 12-well Transwells® (TWs, Corning, 0.4μm, 12 well, polystyrene plates) plates in 2 mL media. After overnight attachment, media was replaced and 1 × 10^5^ MM cells (RPMI-8226, AMO1 or U266) were added to both the stromal layer at the bottom and to the upper chamber of the TW. For mono-culture (MC) controls, MM cells were seeded as indicated for CC, excluding the HS-5 cells. Total media in all cases was 3 mL/well.

### Cell viability assays

To assess cell viability in co-culture, cells were cultured as indicated above and incubated with drugs at the indicated concentrations for 24 h. MM cells from TWs were gently resuspended, collected by centrifugation and washed with cold PBS (both at 5 min, 1400 rpm, 4 °C). Then MM cells were resuspended in 120 μL PBS and transferred to a 96-well V-bottom plate (Corning). After another wash with cold PBS (3 min, 1400 rpm, 4 °C), cells were stained with 100 μL fixable viability dye eFluor™ 506 (eBioscience, #65–0866-14, 1:1000 in cold PBS) for 30 min on ice. Cells were washed with cold PBS (3 min, 1400 rpm, 4 °C) and fixed in 100 μL of 1% paraformaldehyde and subjected to flow cytometry analysis. Unstained and positive-death controls were included to define viability gates. Data were acquired on a CytoFLEX cytometer (FSC = 35, SSC = 40; detection at 525/40 nm using the 405 nm laser) and analyzed using FlowJo software.

To assess cell viability in monocultures, MM cells were seeded in triplicate at a density of 1 × 10^5^ cells/mL in 96-wells plates and incubated with drugs/soluble factors at the indicated times and concentrations. Cell viability was assessed by adding 50 μM resazurin to each well, followed by measurement of absorbance at 570 nm and 600 nm after 4 h using a SPECTROstar Nano microplate reader (BMG Labtech). Viability was calculated by subtracting background absorbance at 600 nm from the 570 nm signal.

### Enzyme-Linked Immunosorbent Assay (ELISA)

After 48–72 h of co-culture, media were collected and clarified by centrifugation (1400 rpm, 5 min, RT). IL-6 concentrations were quantified using the Human IL-6 Uncoated ELISA Kit (ThermoFisher, #88–7066-88) and TGF-β concentrations were quantified using the Mouse TGF-β1 DuoSet ELISA kit (R&D systems, # DY1679-05), according to the manufacturer’s instructions. Media samples for TGF-β1 measurement were diluted 1:2.8 and for IL-6, MC samples were undiluted while CC samples were diluted 1:5000 in their respective dilution buffers. Absorbance was measured at 450 nm with background correction at 560 nm using a GloMAx microplate reader (Promega). Standard curves were generated using a four-parameter logistic (4-PL) regression model and cytokine concentrations were calculated using the Arigo ELISA Calculator.

### RNA extraction and Reverse Transcription-Quantitative Polymerase Chain Reaction (RT-qPCR)

After 72 h of co-culture, MM cells were collected from TW inserts by gentle resuspension. Cells from the lower compartment including HS-5 and MM cells were washed with PBS to reduce the amount of MM cells and leave the attaching cells at the bottom. HS-5 and attached MM cells were harvested by trypsinization, collected in tubes and counted using an automatic cell counter. RNA from up to 5 × 10^5^ cells was extracted using the RNeasy® Micro Kit (Qiagen, #74104), following the manufacturer’s protocol. RNA concentration and purity were assessed using a NanoDrop spectrophotometer. Samples with 260/280 ratios of ~2.0 and 260/230 ratios of 2.0–2.2 were used for downstream analysis. cDNA was synthesized from 25 ng RNA using the iScriptTM cDNA Synthesis Kit (BioRad, #1708890), and the resulting cDNA was diluted 1:50 with RNase-free water.

Quantitative PCR (qPCR) was performed on a CFX Connect Real-Time PCR Detection System (BioRad) using iCycler iQ PCR 96-well plates and iQ SYBR Green Supermix (BioRad, #1708880) in a total volume of 15 µL. Thermal cycling conditions were set as follows: 95 °C for 3 min, followed by 40 cycles of 95 °C for 10 s and the primer-specific annealing temperature for 30 s (Supplementary Table 1). After amplification, melt-curve analysis was performed from 65 °C to 95 °C with 0.5 °C increments every 5 s. Data processing was performed using CFX Manager Software (Bio-Rad). Ct values were normalized to reference genes β-actin and RPL19. Relative gene expression (RGE) was calculated as RGE = (E/100) × 2^ΔCt^, where E denotes PCR efficiency.

Primers were selected using PrimerBank, with criteria for amplification size (70–200 base pairs), primer length (18–30 bases), GC content (35–65%), and specificity, confirmed by Primer-Blast. Primers targeting α-SMA, β-actin, FAP, FGF7, IL-6, and RPL-19 were synthesized by IDT and dissolved in RNase-free water at 100 pmol/µL. Primer sequences and annealing temperatures are listed in Supplementary Table 1.

### Liquid chromatography - mass Spectrometry (LC-MS)-based metabolomics

For all metabolomics assays, cells were cultured in Dulbecco’s Modified Eagle Medium (DMEM) without glucose, glutamine and phenol red (Gibco, # A1443001), supplemented with 25 mM [U-^13^C]glucose (Cambridge Isotopes, #CLM-1396-MPT-1), 2 mM L-glutamine (Gibco, #25030081), 10% fetal bovine serum (FBS) (Gibco, #16000044) and 100 μg/mL penicillin/streptomycin (Gibco, #15140122). ^13^C-tracer experiments were performed as described [9, 34], with minor changes. Three biological replicates were analyzed for each condition. For co-culture experiments, HS-5 cells were seeded as indicated above in RPMI-1640 medium overnight, after which media were replaced with metabolomics media and MM cells were also seeded as described above in this metabolomics media. After 48 h of co-culture, MM cells from TWs were gently resuspended, collected by centrifugation for 5 min at 1000*g* at 4 °C and washed with ice-cold PBS. Metabolites were extracted by adding 50 μL of ice-cold extraction buffer (methanol/acetonitrile/water, 2:2:1) to the cell pellets. For mono-culture experiments, MM cells were seeded at a density of 2 × 10^5^ cells/mL in 12-well plates and cultured with IL-6 at the specified concentrations. After 48 h, cells were harvested as described above. For extracellular metabolites, media samples were collected prior to harvesting. Metabolites were extracted by diluting 10 μL medium in 1 mL of the same extraction buffer. All samples were shaken for 10 minutes at 4 °C, centrifuged at 14.000 g for 15 minutes at 4 °C and supernatants were collected for LC-MS analysis.

LC-MS analysis was performed on a Q-Exactive HF mass spectrometer (Thermo Fisher Scientific) coupled to a Vanquish autosampler and pump (Thermo Fisher Scientific). Chromatographic separation was performed on a ZIC-pHILIC column (2.1 × 150 mm, 5 μm; Merck) with a corresponding guard column (2.1 × 20 mm, 5 μm; Merck) using a linear gradient of acetonitrile and a buffer containing 20 mM (NH_4_)_2_CO_3_ and 0.1% NH_4_OH in ULC/MS grade water. Flow rate was set at 100 μL/min. The MS operated in polarity-switching mode with spray voltages of +4.5 kV and −3.5 kV at 120,000 resolution. Peak areas were integrated using TraceFinder (Thermo Fisher Scientific). Metabolites were identified on the basis of exact mass (±5 ppm) and matched retention times to authenticated standards. Intracellular metabolite abundances were normalized to the mean intensity across all detected metabolites per sample. Isotopologue distributions were corrected for natural abundance of ^13^C.

### Integrative analysis of proteomics and cytokine datasets

Proteome and cytokine datasets were retrieved from the MYELOMICS resource described by Kropivsek et al. (2023) [35]. The proteomics dataset consisted of normalized protein abundance for 5.663 unique proteins measured in CD138^+^ plasma cells from 76 MM patients. The cytokine dataset comprised concentrations of ~ 64 cytokines quantified in bone marrow serum from 48 MM patients. For downstream analysis, only MM patients (excluding post-allo-transplant controls) with paired proteome and cytokine data were included. Data were filtered for metabolic enzymes based on a curated gene list derived from KEGG. Protein and cytokine values were Z-score normalized and correlations were calculated to assess associations between cytokine and metabolic protein levels per patient.

### Statistical analysis

All data are presented as mean ± standard deviation (SD) unless stated otherwise. Statistical analyses and graphing were performed in R (version 4.5.1) using RStudio. Two-sided Welch’s t-tests (unequal variance) were applied to evaluate differences between groups, with a significance threshold of *p* < 0.05 (* = *p* ≤ 0.05, ** = *p* ≤ 0.01, *** = *p* ≤ 0.001). Pearson correlation coefficients (R) were calculated to assess relationships between variables. Heatmaps were generated using hierarchical clustering with Euclidean distance and Ward.D2 linkage. Dose-response curves for cell viability were fitted with a three-parameter log-logistic model.

## Results

### BMSC co-culture drives metabolic rewiring in a heterogeneous panel of MM cells.

**Fig. 1:**
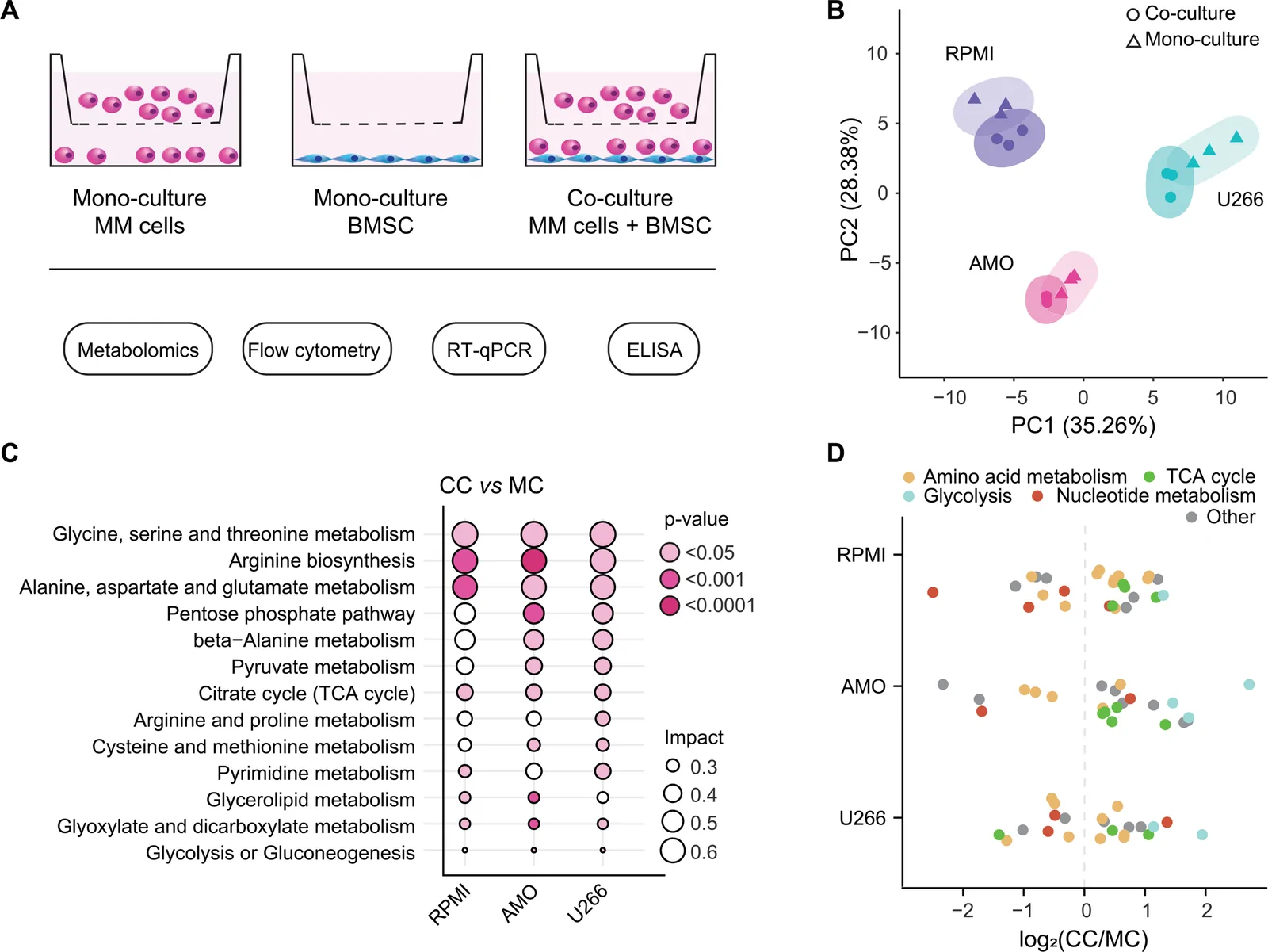
Co-culture drives metabolic rewiring in a heterogeneous panel of MM cells. **A)** Schematic overview of the experimental set-up used for co-culture experiments. Mono-culture (MC) conditions consisted of MM cells (RPMI-8226, AMO1 or U266) placed in the bottom chamber and in the Transwell (TW) insert, or HS-5 BMSCs seeded in the bottom chamber with an empty TW insert. Co-culture (CC) conditions consisted of HS-5 cells and MM cells in the bottom chamber with MM cells in the TW insert. **B-D)** Analysis of LC-MS-based metabolomics data derived from [U-^13^C]glucose tracing in RPMI-8226, AMO1 or U266 cells in MC or CC with HS-5 for 48 h (*n* = 3 replicates cultured in parallel wells). Total metabolite levels were calculated as the sum of all isotopologues per metabolite. **B)** Principal component analysis (PCA) of total metabolite levels in RPMI-8226, AMO1 or U266 cells in MC (triangles) or CC (dots). **C)** Pathway enrichment analysis comparing total metabolite levels in CC and MC conditions across cell lines. Results were filtered for pathways with >2 compound hits, *p* < 0.05, and pathway impact > 0.24 in at least one cell type. Enriched pathways are shown as bubble plot, where point size indicates pathway impact and color denotes *p*-values. Larger and darker points represent pathways more strongly perturbed in CC vs MC conditions. **D)** Volcano plots showing differential metabolite abundances in RPMI-8226, AMO1 or U266 cells comparing CC vs MC. Mean metabolite levels were compared by Student’s t-test and metabolites with *p* ≤ 0.05 are plotted. Points are colored by metabolic pathway

We previously showed that co-culture of RPMI-8226 MM cells with BMSCs induces metabolic rewiring in MM cells, linked to BTZ resistance [6]. To investigate whether BMSCs induce similar metabolic remodeling in a heterogeneous panel of MM cells, we performed LC-MS-based metabolomics on RPMI-8226, AMO1 and U266 MM cells after 48 h of co-culture with HS-5 BMSCs in the presence of [U-^13^C]glucose. Genetically, AMO1 and U266 cells are characterized by the (t12;14) and the most frequently occurring (t11;14) translocation, respectively, while RPMI-8226 cells are characterized by a (t14;16) translocation [32, 33, 36]. To better recapitulate the complexity of the TME with both direct and indirect contacts between BMSCs and MM cells, we adapted the model of Dziadowicz et al. [37] for metabolomics studies. In this model, MM cells are seeded in direct contact with HS-5 BMSCs in the bottom chamber, while additional MM cells are cultured in the upper transwell (TW) insert (Fig. 1A), allowing for rapid isolation of MM cells from TW inserts for metabolic profiling.

Focusing on total metabolite levels, principal component analysis (PCA) revealed two key features. Each MM cell line displayed a distinct metabolic profile, reflecting cellular heterogeneity, and co-culture with BMSCs induced clear metabolic shifts in every line (Fig. 1B). Pathway enrichment analysis based on total metabolite levels revealed that co-culture induced a significant and consistent metabolic rewiring across all MM cell lines (Fig. 1C). The most significantly enriched pathways in all cell lines related to amino acid metabolism (e.g., glycine and serine metabolism and arginine metabolism) and the TCA cycle (e.g., pyruvate metabolism and citrate cycle) (Fig. 1C), indicating altered energy and biosynthetic demands in co-cultured MM cells. In two out of three cell lines, additional enrichment was observed in the pentose phosphate pathway (PPP) and nucleotide metabolism, pathways linked to a proliferative state. At the single metabolite level, 8 metabolites were significantly up- or downregulated between co-culture and mono-cultured MM cells across all cell lines, including serine, TCA cycle intermediates α-ketoglutarate and cis-aconitate, and the glycolytic end-product lactate (Supplementary Fig. 1A). Because pathway enrichment can arise from either increased or decreased metabolite levels, and because different metabolites may drive pathway enrichment in different cell lines, we compared the relative abundance of individual metabolites in the top-scoring pathways in co-cultured *versus* mono-cultured cells (Fig. 1D). This analysis showed a consistent upregulation of both glycolytic and TCA cycle intermediates in co-cultured MM cells, indicative of increased glycolysis and mitochondrial activity. In contrast, while nucleotides and amino acids were consistently changed between mono- and co-cultured cells, their levels were both up- and downregulated (Fig. 1D).

**Fig. 2:**
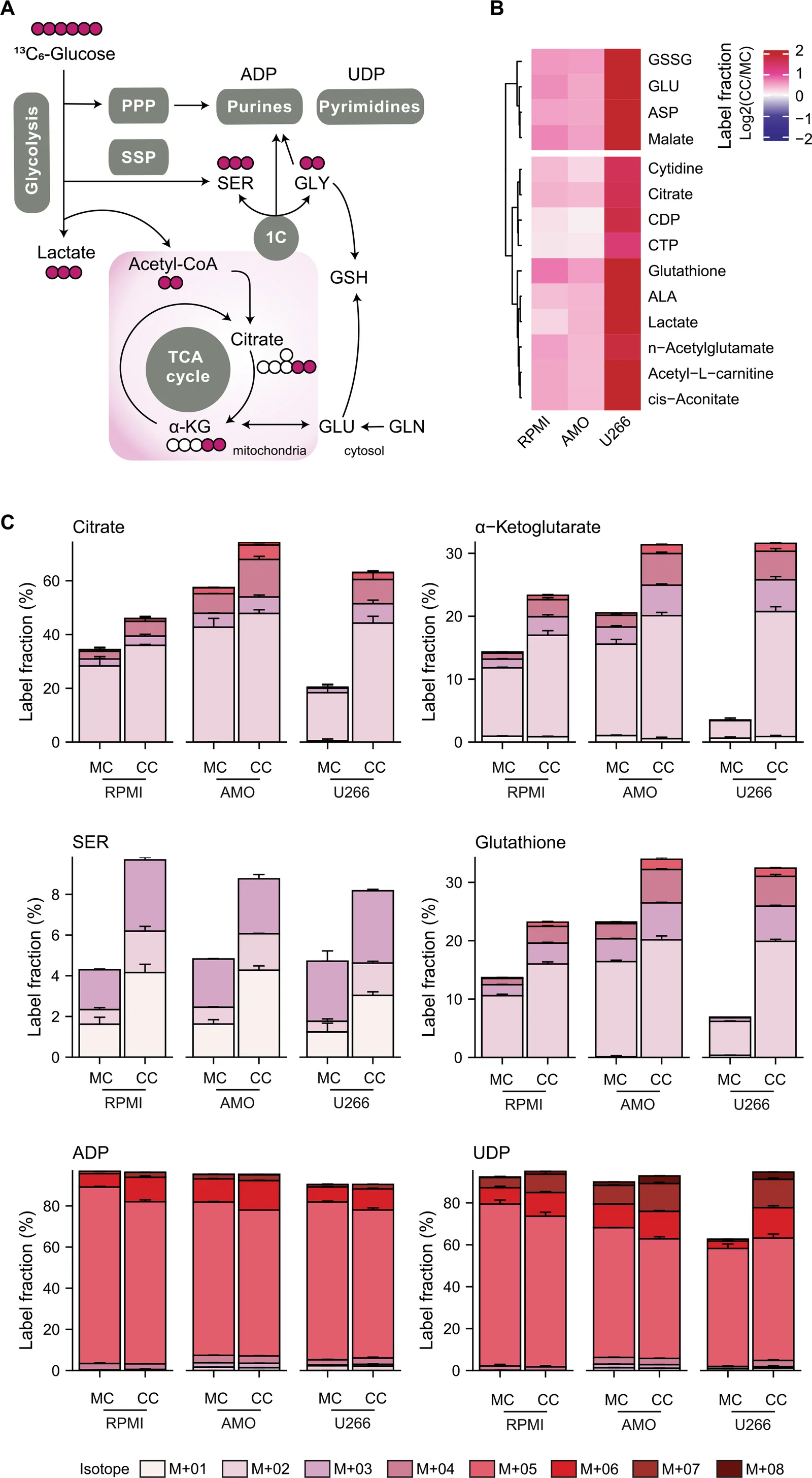
Co-culture promotes a metabolic shift towards a bortezomib-resistant phenotype in MM cells. **A)** Schematic overview of the [U-^13^C]glucose isotope tracing approach, illustrating the fate of ^13^C through glycolysis and associated pathways. Glucose-derived carbon generates pyruvate via glycolysis, which can be converted into lactate or oxidized in the TCA cycle via acetyl-CoA to support mitochondrial respiration and biosynthesis. In addition, glycolytic intermediates feed into the pentose phosphate pathway (PPP) to support redox balance and nucleotide synthesis, and the serine synthesis pathway (SSP) to support one-carbon (1C) metabolism, nucleotide synthesis, and antioxidant capacity. Glucose-derived labeled carbons are depicted as colored circles, and unlabeled carbons as open circles. **B)** Heatmap showing log_2_ fold changes (FC) in ^13^C-label incorporation in CC vs MC cells of metabolites consistently upregulated across RPMI-8226, AMO1, or U266 cells after 48 h with [U-^13^C]glucose (*n* = 3). Fractional ^13^C enrichment was calculated for each metabolite by the sum of peak intensities of all labeled isotopologues divided by the total peak intensity. **C)** Fractional ^13^C enrichment and labeled isotopologue patterns of citrate, α-ketoglutarate, serine, glutathione and nucleotides (UDP and ADP) in RPMI-8226, AMO1, or U266 cells under MC and CC conditions after 48 h with [U-^13^C]glucose. Data are presented as mean ± SD (*n* = 3). Different colors indicate different isotopologues

To investigate the metabolic rewiring under co-culture conditions in more depth, we analyzed the incorporation of labeled carbon from [U-^13^C]glucose into key metabolic pathways. In addition to fueling glycolysis and the TCA cycle, glucose also supplies key intermediates to the PPP and SSP, thereby supporting mitochondrial respiration, redox balance, nucleotide and one-carbon metabolism (Fig. 2A). Analysis of glucose-derived carbon incorporation into these pathways showed that glucose-derived carbon was incorporated in 69 metabolites (Supplementary Fig. 1B). Compared with mono-culture, co-culture consistently enhanced ^13^C incorporation into 14 metabolites across all lines, spanning pathways such as glycolysis (lactate), nucleotide biosynthesis (CDP, CTP, cytidine), the TCA cycle (citrate, malate, cis-aconitate) and redox metabolism (glutathione, GSSG) (Fig. 2B), indicating that co-culture broadly elevates these pathway activities. Higher levels of ^13^C-labeled lactate [M+3] together with higher total lactate levels across cell lines (Supplementary Fig. 1C) indicate enhanced glucose-derived carbon incorporation into glycolytic intermediates and end products after co-culture. Similarly, co-cultured MM cells showed increased incorporation of glucose-derived carbon into TCA cycle intermediates, reflected by a higher abundance of [M+3], [M+4] and [M+5] isotopologues (Fig. 2C), together with higher total levels of these metabolites (Supplementary Fig. 1C). Together, these findings indicate greater glucose oxidation through acetyl-CoA and enhanced mitochondrial activity.

Pathways branching off from glycolysis, including the SSP and nucleotide synthesis, also showed increased glucose-derived carbon incorporation under co-culture conditions. Under mono-culture conditions, only low levels of ^13^C incorporation in serine were observed (Supplementary Fig. 1C), consistent with limited *de novo* serine synthesis in the presence of exogenous serine. Co-culture with HS-5 markedly increased the fraction of ^13^C-labeled serine in all cell lines (Fig. 2C). Detailed isotopologue analysis revealed higher fractions of fully labeled serine [M+3], derived directly from glucose via the SSP, together with serine [M+1], indicative of serine-glycine cycling and active one-carbon metabolism (Fig. 2C). Although intracellular serine pools were reduced following co-culture (Supplementary Fig. 1C), the increased labeling suggests enhanced incorporation of glucose-derived carbon into serine metabolism, likely reflecting rapid utilization in downstream pathways. In line with this, co-culture increased the incorporation of glucose-derived carbon into glutathione (Fig. 2C, Supplementary Fig. 1C), indicating altered glutathione metabolism involving both glutamate- and glycine-derived carbon. While total glutathione pools were reduced in some cell lines, this likely reflects increased glutathione turnover and utilization under co-culture conditions. Finally, nucleotide intermediates (ADP, UDP) showed enrichment of higher-mass isotopologues [*M*>+5] in co-cultured cells, indicative of increased *de novo* purine and pyrimidine synthesis (Fig. 2C). Together, these data demonstrate that co-culture with HS-5 cells induces a conserved metabolic rewiring across MM cell lines, characterized by enhanced incorporation of glucose-derived carbon into the TCA cycle, SSP, glutathione metabolism and nucleotide biosynthesis. These metabolic adaptations overlap with pathways previously associated with BTZ resistance [8, 9], suggesting that stromal cells play a role in shaping MM metabolism and potentially driving metabolism-mediated drug resistance. Importantly, these metabolic adaptations are unlikely to be explained by extracellular nutrient depletion. Analysis of extracellular media showed that, although consumption of critical metabolites such as glucose, serine and glutamine increased during co-culture due to the higher number of cells, substantial amounts of extracellular nutrients remained available at the experimental endpoint (Supplementary Fig. 1D).

**Fig. 3:**
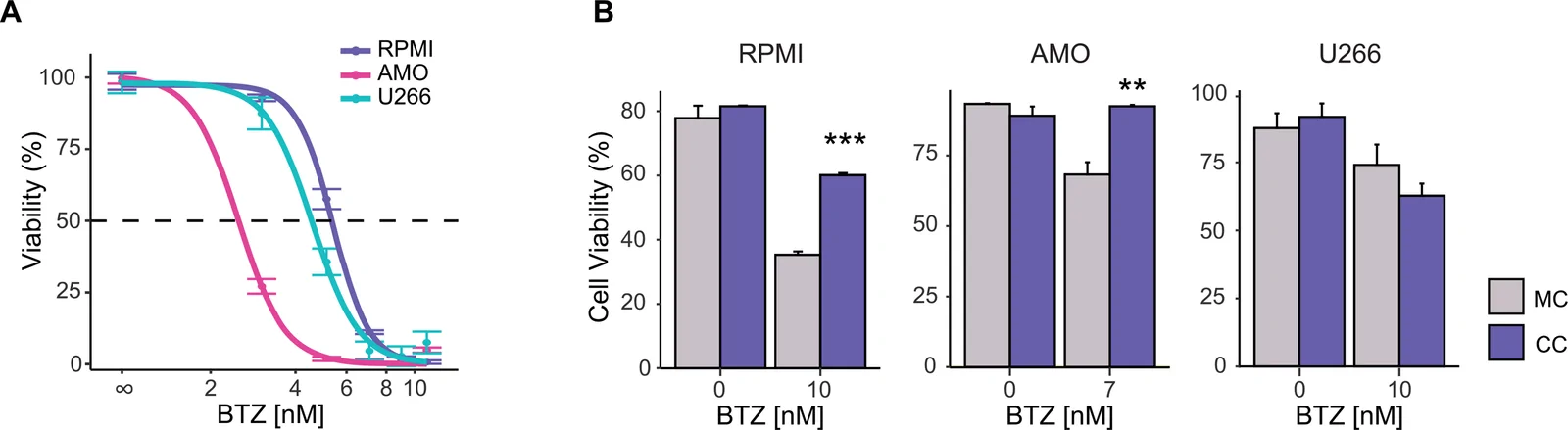
Co-culture reduces bortezomib sensitivity in multiple myeloma cells. **A)** Dose-response curves of RPMI-8226, AMO1 and U266 cells treated for 48 h with increasing concentrations of bortezomib (BTZ). Results represent cell viability compared to non-treated controls (%). **B)** Cell viability of RPMI-8226, AMO1 and U266 cells in mono-culture (MC) or co-culture (CC) conditions in the absence or presence of 7–10 nM BTZ for 24 h. Data are presented as mean ± SD (*n* = 3 replicates cultured in parallel wells). *p* values were calculated using Student’s t-test. **: *p* ≤ 0.01; ***: *p* ≤ 0.001

Because several of the metabolic adaptations induced by co-culture overlap with metabolic features previously associated with reduced sensitivity to the proteasome inhibitor BTZ in MM cells [6, 8, 9], we examined whether co-culture impacted BTZ response in these cell lines. As expected, intrinsic sensitivity to BTZ varied between the three MM cell lines, with AMO1 being the most sensitive (IC_50_ = 2.5 ± 0.14 nM), followed by U266 (IC_50_ = 4.48 ± 0.12 nM) and RPMI-8226 (IC_50_ = 5.23 ± 0.43 nM) (Fig. 3A). Co-culture significantly reduced BTZ sensitivity in RPMI-8226 and AMO1 cells, whereas U266 cells were unaffected (Fig. 3B), indicating that the impact of stromal cues on drug response is cell line-dependent. Together, these data demonstrate that co-culture with HS-5 cells induces a broadly conserved metabolic remodeling across genetically distinct MM cell lines. In two of the three cell lines (RPMI-8226 and AMO1), this remodeling was accompanied by reduced BTZ sensitivity, whereas U266 cells remained insensitive to the effects of co-culture on drug response, highlighting the contribution of additional cell-intrinsic determinants of BTZ sensitivity.

### Co-culture induces stromal activation with CAF-like features and creates a cytokine-rich microenvironment

**Fig. 4:**
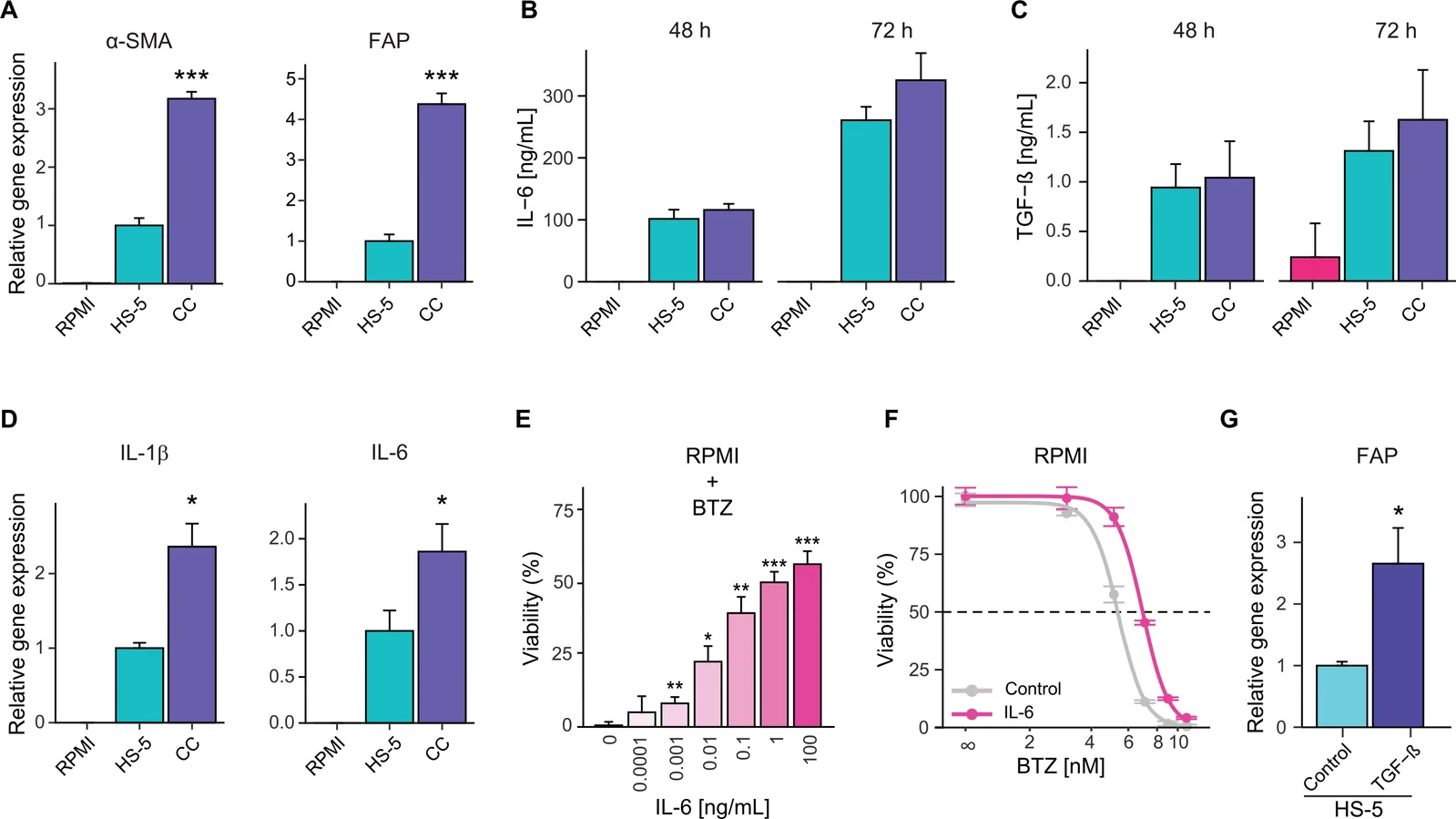
Co-culture drives stromal activation with CAF-like features in stromal cells and creates a cytokine-rich, pro-inflammatory environment. **A)** Relative gene expression of α-SMA and FAP in RPMI-8226 and HS-5 cells after 72 h of mono-culture (MC) or co-culture (CC). Data are presented as mean ± SD relative to HS-5 (*n* = 3 replicates cultured in parallel wells). **B-C)** IL-6 **(B)** and TGF-β **(C)** levels measured by ELISA in conditioned media collected after 48 h and 72 h from RPMI-8226 MC, HS-5 MC or CC conditions. Data are presented as mean ± SD (*n* = 4). **D**) Relative gene expression of IL-1β and IL-6 in RPMI-8226 and HS-5 cells after 72 h of MC or CC. Data are presented as mean ± SD (*n* = 3). **E)** Cell viability of RPMI-8226 cells treated with 5 nM BTZ for 48 h ± IL-6 (0–100 ng/mL). Data were normalized to untreated controls and presented as mean ± SD (*n* = 3). **F)** Dose-response curve of RPMI-8226 cells ± IL-6 (100 ng/mL) treated with BTZ. Cells were pre-treated with IL-6 for 24 h, followed by BTZ treatment for 48 h with the indicated concentrations. Results represent cell viability (%) compared to untreated controls (mean ± SD, *n* = 3). **G)** Relative gene expression of FAP in HS-5 cells treated with 0 or 2.5 ng/mL TGF-β for 48 h. Data are represented as mean ± SD (*n* = 3). *p* values were calculated by Student’s t-test. *: *p* ≤ 0.05**: *p* ≤ 0.01,***: *p* ≤ 0.001

We next sought to characterize the stromal compartment of our co-culture model to identify potential drivers of the metabolic rewiring observed in MM cells. BMSCs from MM patients are known to acquire a CAF-like phenotype, characterized by markers such as α-SMA and FAP, and this phenotype has been associated with disease progression and reduced BTZ sensitivity in MM [22, 23]. To determine whether stromal cells acquire similar CAF-like features in our model, we quantified α-SMA and FAP expression in mono-cultures of RPMI-8226 and HS-5 cells, as well as in the bottom layer of our co-culture, which contains both cell types (Fig. 1A). As expected, MM cells alone showed no detectable expression of α-SMA or FAP, consistent with these markers being predominantly expressed by BMSCs [23, 38] (Fig. 4A). In contrast, co-cultured cells exhibited significant upregulation of α-SMA and FAP compared to HS-5 mono-cultures (Fig. 4A), indicating that interaction with MM cells induces stromal activation with CAF-like features.

In addition to stromal activation, the MM microenvironment is typically characterized by unbalanced levels of both pro- and anti-inflammatory cytokines that promote tumor progression and therapy resistance [39]. Both IL-6 and TGF-β have previously been reported to accumulate in MM-BMSC co-cultures, with their levels frequently showing a strong correlation [40–42]. Consistent with these observations, analysis of conditioned cell culture media at 48 and 72 h showed that both IL-6 and TGF-β were secreted by HS-5 mono-cultures and co-cultures, with levels increasing over time (Fig. 4B–C). RPMI-8226 mono-cultures did not secrete detectable IL-6 and produced only low levels of TGF-β at later time points, as previously described [43, 44] (Fig. 4B–C). Increased IL-6 secretion was accompanied by upregulated expression of IL-6 and IL-1β in HS-5 cells (Fig. 4D), the latter being a known upstream regulator of IL-6 production [45, 46]. Both IL-6 and IL-1β expression were further increased under co-culture conditions (Fig. 4D), whereas RPMI-8226 cells alone expressed little to no IL-1β and IL-6 (Fig. 4D), consistent with previous reports [47]. Similar IL-6 secretion patterns were observed in co-culture with AMO1 and U266 cells (Supplementary Fig. 2A), indicating that stromal cells are the primary source of IL-6 in our co-culture system.

Given the increased secretion of IL-6 and TGF-β under co-culture conditions, we next investigated whether these cytokines contribute to the co-culture phenotype. IL-6, a key pro-inflammatory cytokine constitutively secreted by BMSCs [30, 48], is linked to BTZ resistance and poor prognosis in MM [49–51], while TGF-β is a key inducer of fibroblast activation across cancers [21, 22], and is elevated in MM [52–54]. We therefore hypothesized that TGF-β may contribute to the activation of BMSCs in our model, whereas IL-6 could mediate reduced sensitivity towards BTZ in co-cultured MM cells. To test this, RPMI-8226 cells were treated with increasing concentrations of recombinant IL-6 in the presence of 5 nM BTZ. While BTZ alone resulted in an almost complete loss of cell viability, IL-6 significantly rescued viability in a dose-dependent manner, with >50% rescue at 100 ng/mL IL-6 (Fig. 4E), a concentration comparable to that measured in our co-culture medium. Although this concentration exceeds circulating IL-6 levels typically reported in MM patients, it was selected to mimic the sustained local exposure observed in our co-culture system rather than systemic cytokine concentrations. Importantly, reduced BTZ sensitivity was already observed at substantially lower IL-6 concentrations, including concentrations of 5–300 pg/mL, which overlap with those reported in MM patients [55, 56] (Fig. 4E). Moreover, IL-6 shifted the BTZ IC_50_ of RPMI-8226 cells by 1.58 nM (Fig. 4F). Similar IL-6-mediated protection against BTZ was observed in AMO1 cells and to a lesser extent in U266 cells (Supplementary Fig. 2B), consistent with their differential responses in co-culture. To investigate the role of TGF-β in stromal activation, HS-5 mono-cultures were treated with 2.5 ng/mL TGF-β, corresponding to the concentration detected after 72 h of co-culture. TGF-β treatment increased FAP expression (Fig. 4G), but did not induce α-SMA expression (Supplementary Fig. 2C). These findings indicate that TGF-β induces selected features of stromal activation but is not sufficient to reproduce the broader CAF-like phenotype observed in co-culture, suggesting that additional stromal or tumor-derived signals contribute to this activation.

Together, our findings demonstrate that the MM-HS-5 co-culture model effectively reproduces key features of the MM TME, including stromal activation with CAF-like characteristics, increased cytokine production and reduced BTZ sensitivity in MM cells. Our data further support distinct roles for stromal-derived cytokines, with IL-6 contributing to reduced BTZ sensitivity and TGF-β contributing to stromal activation.

### IL-6 recapitulates key aspects of the co-culture-induced metabolic phenotype

**Fig. 5:**
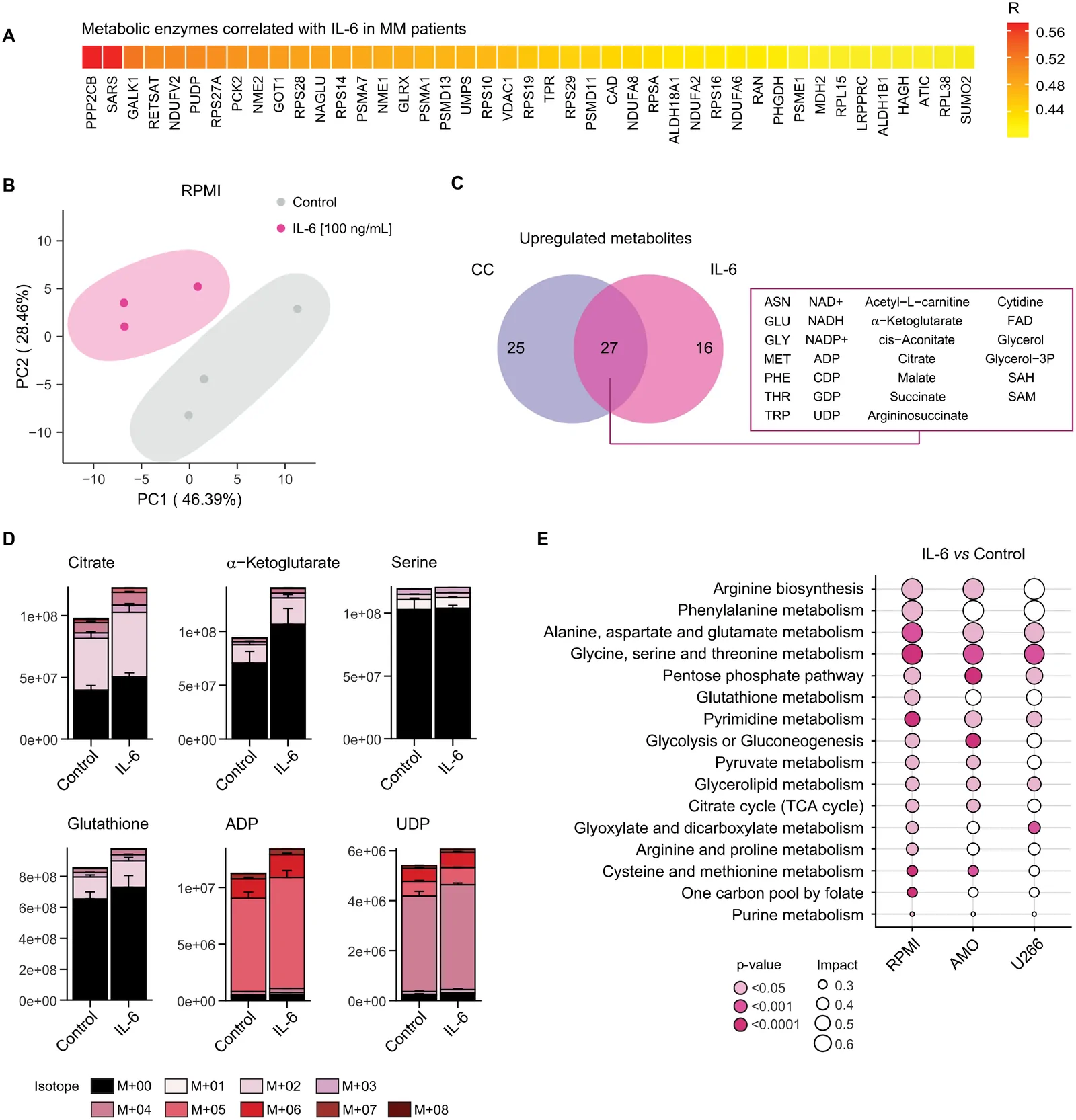
IL-6 recapitulates key aspects of the co-culture-induced metabolic phenotype. **A)** Heatmap showing metabolic proteins whose abundances correlate positively with IL-6 cytokine levels (Pearson’s *R* > 0.4) in CD138+ plasma cells from MM patients in the MYELOMICS cohort [35]. Color encodes the correlation coefficient. **B)** Principal component analysis (PCA) of total metabolite levels comparing treatment with 100 ng/mL IL-6 versus no IL-6 for 48 h (*n* = 3) in RPMI-8226 cells. **C)** Venn diagram showing the upregulated metabolites in RPMI-8226 after co-culture with BMSCs or IL-6 stimulation, as compared to respective controls (*n* = 3 ). Data were obtained from 2 independent experiments. The overlapping region highlights metabolites that were consistently upregulated (log2 fold change > 0.05) under both conditions, which are listed below the diagram. **D)** Bar graphs showing peak areas of all isotopologues of TCA cycle intermediates (citrate, α-ketoglutarate), serine, glutathione and nucleotides (UDP and ADP) in RPMI-8226 cells treated with or without 100 ng/mL IL-6 in the presence of [*U*-^13^C]glucose for 48 h. Data are presented as mean ± SD (*n* = 3). Different colors indicate different isotopologues. **E)** Pathway enrichment analysis of RPMI-8226, AMO1 and U266 cells treated for 48 h with 100 ng/mL IL-6 versus untreated controls (*n* = 3). Results were filtered for pathways with >2 compound hits and pathways with a *p* < 0.05 and impact > 0.24 are shown in pink

Considering the impact of BMSC-derived IL-6 on BTZ response, we next asked whether IL-6 also contributes to the metabolic reprogramming observed in MM cells. To determine whether the metabolic associations identified in our experimental model are also reflected in patients samples, we analyzed the MYELOMICS proteomics dataset [35] and examined correlations between IL-6 abundance and metabolic protein abundance. We applied a threshold of *r* > 0.4 to identify biologically meaningful correlations, representing moderate-to-strong correlations within this patient cohort. Analysis of the MYELOMICS dataset revealed that IL-6 abundance positively correlated with proteins involved in mitochondrial respiration (e.g., NDUFV2, NDUFA6, NDUFB4), redox regulation (e.g., ALDH18A1, GLRX, MDH2), nucleotide metabolism (e.g., ATIC, UMPS) and glycolysis-linked or serine-linked metabolism (e.g., PHGDH, PCK2) (Fig. 5A). In addition, numerous ribosomal proteins (e.g. RPS27A, RPL15, RPS10, RPS11) demonstrated positive correlations with IL-6, suggesting increased abundance of the translational machinery in IL-6-rich patient samples (Fig. 5A). Collectively, these data demonstrate that higher IL-6 abundance in MM patients is associated with increased expression of proteins involved in metabolic and biosynthetic pathways, supporting the clinical relevance of the associations in our experimental models. In contrast to IL-6, TGF-β1 correlated with only a small set of metabolic proteins (Supplementary Fig. 2D), including MED1 and TYMP (nucleotide metabolism), and CYP27A1, DPYD, and NCOA2 (lipid, pyrimidine, and cofactor metabolism, respectively). Compared with IL-6, TGF-β1 therefore exhibited a much weaker association with metabolic protein expression in patient samples.

To determine whether IL-6 could recapitulate the metabolic phenotype induced by stromal co-culture, RPMI-8226 cells were treated with 100 ng/mL recombinant IL-6 for 48 h in the presence of [U-^13^C]-glucose. PCA revealed a clear separation between IL-6-treated and untreated cells based on their metabolite profiles, indicating that IL-6 induces substantial metabolic changes (Fig. 5B). To assess the extent to which IL-6 contributes to the co-culture-induced metabolic phenotype, we compared metabolites significantly upregulated in both conditions. 27 metabolites were significantly increased in both IL-6-treated and co-cultured RPMI-8226 cells (Fig. 5C), including amino acids (glutamate, glycine), TCA cycle intermediates (citrate, α-ketoglutarate), redox cofactors (NAD(P)+, NADH) and nucleotides (ADP, UDP) (Fig. 5D). In contrast to the pronounced labeling changes observed in co-culture, IL-6 treatment produced only modest alterations in isotopologue distributions of representative metabolites, including citrate, α-ketoglutarate, serine, glutathione, ADP and UDP, despite increases in their total intracellular levels (Fig. 5D). To determine whether these observations extended beyond RPMI-8226 cells, we repeated the metabolic analysis including AMO1 and U266 cells. PCA similarly demonstrated separation between IL-6-treated and control AMO1 cells, whereas only limited separation was observed in U266 cells, indicating a weaker metabolic response to IL-6 (Supplementary Fig. 2E). Pathway enrichment analysis revealed substantial overlap across all three cell lines, particularly for serine-glycine metabolism, the PPP and nucleotide metabolism (Fig. 5E), indicating conserved metabolic effects of IL-6. Like RPMI-8226, AMO1 shared a larger number of significantly increased metabolites with the co-culture conditions than U266 (Supplementary Fig. 2F), consistent with the stronger metabolic response to IL-6 in those cell lines. Nevertheless, key metabolites, including citrate, α-ketoglutarate and nucleotides overlapped between IL-6 treatment and co-culture in all three cell lines. Finally, isotopologue analysis demonstrated only modest changes in glucose-derived carbon incorporation following IL-6 treatment, particularly in U266 cells, indicating that IL-6 reproduces metabolite pool changes more strongly than the glucose-labeling patterns observed during co-culture (Supplementary Fig. 2G).

Together, these findings demonstrate that IL-6 recapitulates key aspects of the metabolic remodeling induced by BMSC co-culture, particularly in RPMI-8226 and AMO1, including increased TCA cycle activity, nucleotide synthesis, and serine-related metabolism. Combined with the protein correlations observed in patient samples, these data support a role for IL-6 as an important contributor to the metabolic adaptations associated with stromal interaction and reduced BTZ sensitivity, while indicating that additional microenvironmental factors participate in shaping the complete co-culture phenotype.

## Discussion

MM depends on extensive metabolic remodeling to support malignant growth and drug resistance. The metabolic rewiring of MM cells appears to be shaped by their interaction with components within the TME [6], yet the extent and precise nature of the TME-induced metabolic rewiring and the specific stromal signals that drive these adaptations remain incompletely defined. By combining a metabolomics-compatible co-culture system with stable isotope-tracing metabolomics, we here demonstrate that BMSCs induce a convergent metabolic phenotype in a heterogeneous panel of MM cells. Despite their intrinsic differences, all MM cells co-cultured with BMSCs displayed increased activity of the serine synthesis pathway, amino acid metabolism, nucleotide biosynthesis and the TCA cycle. These changes closely resemble the metabolic phenotype previously described in BTZ-resistant MM cells by us and others [8, 9, 11, 57], which is characterized by high mitochondrial activity, glycolytic remodeling, induction of the SSP and antioxidant adaptation. These findings suggest that co-culture with BMSCs induces metabolic remodeling that overlaps with metabolic adaptations associated with BTZ resistance.

Consistent with this, the metabolic rewiring induced by co-culture was accompanied by reduced BTZ sensitivity in RPMI-8226 and AMO1 cells, while co-culture did not protect U266 from BTZ toxicity in our co-culture system. The pronounced metabolic remodeling observed in U266 therefore indicates that these adaptations are not, by themselves, sufficient to determine BTZ response in MM cells. Instead, the impact of the TME on drug sensitivity seems to be cell line-depended and influenced by additional cell-intrinsic determinants. Sensitivity to proteasome inhibition is determined not only by metabolism but also by factors such as proteotoxic stress and unfolded protein response capacity. Together, these observations suggest that metabolic remodeling represents one important component of stromal-mediated drug adaptation, but that its contribution to BTZ sensitivity depends on the intrinsic biology of individual MM subtypes.

Characterization of our model system showed that BMSCs acquire an activated state with CAF-like features in response to MM co-culture, marked by increased α-SMA and FAP expression. CAF markers have been previously observed in MM co-culture [23] and in MM patients [22, 58] and are linked to disease progression and BTZ resistance [24, 25]. Factors contributing to CAF induction include cytokines, growth factors, exosomes and direct cell contact [20, 59]. Of these factors, TGF-β, an established CAF inducer [22], was secreted by stromal cells in our co-culture system. While TGF-β increased expression of the CAF-associated marker FAP, it did not reproduce the broader stromal phenotype observed during co-culture. Prior studies have also reported variable effects of TGF-β on α-SMA and FAP [22, 60], highlighting that although TGF-β contributes to stromal reprogramming, CAF activation is multifactorial and cell-type dependent. Importantly, CAFs are recognized as a highly heterogeneous population rather than a single, uniform cell type [61]. Comprehensive characterization incorporating additional molecular markers, transcriptomic profiling and functional assays would be required to fully define CAF identity and heterogeneity in the MM bone marrow microenvironment. However, these findings indicate that stromal activation in MM is multifactorial and cannot be explained by TGF-β alone.

CAFs can induce MM drug resistance through several mechanisms, including modulation of the extracellular matrix [62], cell adhesion-mediated mechanisms [26, 63] or via the secretion of cytokines [64]. As we analyzed drug sensitivity and metabolic rewiring only in MM cells that were grown separately in Transwell inserts, we assumed that soluble factors such as cytokines were responsible for the observed effects on BTZ response and metabolism in MM cells. Several cytokines have previously been linked to BTZ response, including IL-6, IL-8, IL-17, NF-_K_B, SDF-1/CXCL12, and APRIL [27, 65–70]. Of these, IL-6 is widely recognized as a prominent driver of MM drug resistance and poor prognosis [49, 51] and known to be synthesized by stromal cells in high amounts [18, 71]. We indeed observed high levels of IL-6 in our co-culture system, mirroring these findings. IL-6 levels found in co-culture were substantially higher than circulating IL-6 concentrations typically reported in MM patients, with a range of 5–300 pg/mL of IL-6 [55, 56]. The 100 ng/mL recombinant IL-6 concentration was selected to reflect IL-6 levels in our MM-BMSC co-culture system, enabling assessment of whether IL-6 alone could recapitulate the co-culture-induced phenotype.

IL-6 secretion can be induced by direct MM-BMSC contact [72–77], BTZ exposure [71] or upstream regulators such as TGF-β [40, 71] and IL-1β [78]. In our system, both IL-1β and TGF-β were increased in stromal cells upon co-culture, suggesting that multiple stromal cues may work together to enhance IL-6 production. Other factors known to boost IL-6 secretion include other cytokines (e.g., TNF-α [78], IL-2 [79], IL-17 [80], IL-36 [81], IL-32 [82]), exosome uptake [83] or hypoxia [84]. Although these were not directly quantified in our system, many are reported to increase in MM-BMSC co-cultures [64, 85, 86] and may further modulate IL-6 levels.

Functionally, IL-6 reduced MM sensitivity to BTZ in a dose-dependent manner, in RPMI-8226 and AMO1 cells. Moreover, our study shows for the first time that IL-6 is sufficient to induce a metabolic shift in the same MM cells that also mirrors many of the metabolic changes observed in cells in co-culture with BMSCs. IL-6 treatment alone increased total levels of SSP, TCA cycle, nucleotide and antioxidative metabolites, in line with previous studies that have shown that IL-6 modulates metabolism in other cancers [84, 87–90] and increases total levels of serine and SSP enzymes [91], purines [91] and TCA cycle metabolites [88] in other systems. In addition, our analysis of a proteomics dataset of MM patient samples provides an indication that IL-6 is linked to metabolic remodeling in vivo. IL-6 levels were correlated with the levels of metabolic proteins, including components of the mitochondrial respiratory chain, redox-regulating enzymes, and enzymes involved in glycolysis, nucleotide and serine metabolism. These IL-6-associated metabolic signatures in MM patients mirror the observed metabolic rewiring in MM cells during co-culture with BMSCs, positioning IL-6 as a possible regulator of metabolic remodeling in vitro and in vivo. However, we must acknowledge that IL-6 did not recapitulate metabolic or viability effects in U266 cells, meaning that the metabolic effects observed in co-culture are not solely caused by IL-6, and IL-6 does not induce BTZ resistance in these cells. U266 cells have been described to have an autocrine IL-6 loop [92] and differ on IL-6 response depending on surface markers [93], which may explain why they respond differently to exogenous IL-6. If autocrine IL-6 signaling is already active, the addition of IL-6 is unlikely to induce further pathway activation.

We also observed differences between IL-6 treatment and co-culture in their ability to rewire MM metabolism. Co-culture with stromal cells increased both metabolite pool sizes and changed glucose-derived labeling in anabolic pathways, whereas IL-6 alone increased metabolite pools without changing glucose-derived carbon incorporation into glycolytic intermediates and end products. Importantly, changes in steady-state metabolite abundance do not directly reflect metabolic flux. Increased intracellular metabolite pools may arise from multiple processes, including altered nutrient uptake, reduced metabolite consumption, or altered catabolic or anabolic pathway activity. However, extracellular metabolite analysis demonstrated that key nutrients were not depleted during co-culture, suggesting that the observed intracellular remodeling is unlikely to be explained by substrate limitation. Instead, the combined pool size and isotope tracing data indicate coordinated changes in metabolic pathway activity. These findings suggest that IL-6 contributes substantially to this metabolic phenotype induced by stromal cells but does not fully reproduce the complete metabolic remodeling observed during co-culture. One explanation may lie in differences in IL-6 signaling. IL-6 activates either classical signaling through membrane-bound IL-6R or trans-signaling via soluble IL-6R, which activate distinct downstream pathways [66, 88, 94–97]. Previous reports have suggested that IL-6 trans-signaling is elevated in MM patients [98] and in MM-BMSC co-culture [72]. This suggests that stromal co-culture may preferentially activate IL-6 trans-signaling, which may lead to metabolic remodeling that is distinct from remodeling induced by recombinant IL-6 alone. Alternatively, metabolic reprogramming in the MM niche is likely driven by multiple stromal-derived signals acting in concert, rather than IL-6 alone. Metabolic cross-feeding, nutrient competition and other cytokines may all contribute to shaping MM metabolism. Metabolites accumulating in the co-culture medium, such as pyruvate, lactate or glutamate, are suggestive of altered metabolic exchange between stromal and MM cells. Furthermore, CAF-like stromal cells from MM patients are known to secrete additional cytokines (e.g., IGF-1 [22]) that activate pro-survival cascades [21, 99, 100] and may trigger additional metabolic alterations. Future studies using conditioned media, cytokine depletion strategies or stromal-specific IL-6 knockdown will help define the relative contribution of these individual components.

Finally, our findings support therapeutic strategies aimed at disrupting IL-6 signaling in MM. Elevated IL-6 levels in newly diagnosed MM patients correlate with advanced International Staging System (ISS) scores, high β2-microglobulin and increased LDH levels, all markers of poor prognosis. Moreover, the anti-IL-6 antibody Siltuximab [101] and anti-IL-6R antibody Tocilizumab [102] are being tested in clinical trials in combination with proteasome inhibitors. Our data provide mechanistic support for these approaches by demonstrating that IL-6 contributes to metabolic adaptations associated with reduced BTZ sensitivity. However, because IL-6 only partially reproduced the metabolic phenotype induced by stromal co-culture, therapeutic strategies simultaneously targeting additional components of the MM-BMSC interaction may prove effective. For instance, syntenin inhibition has emerged as a promising approach to disrupt BMSC-MM crosstalk and IL-6 secretion by extracellular vesicles, enhancing BTZ sensitivity [103, 104]. Future studies employing stromal-specific IL-6 loss-of-function approaches will be valuable to further define the contribution of stromal-derived IL-6 to metabolic remodeling and therapy response.

We acknowledge that our study has limitations. While our co-culture system recapitulates key features of the myeloma microenvironment, including fibroblast activation, cytokine secretion, metabolic remodeling, and reduced BTZ sensitivity, our findings are based exclusively on in vitro models. These models cannot fully capture the complexity of the bone marrow niche in patients, including immune components, extracellular matrix architecture and oxygen gradients. Second, while our data provide a mechanistic framework linking IL-6-driven metabolism to drug resistance, the therapeutic potential of targeting this axis in patients remains to be determined. To address these gaps, future studies should validate our mechanisms and therapeutic strategies in vivo using patient-derived xenografts and test IL-6/metabolic axis inhibitors (alone and in combination with bortezomib) for efficacy and safety.

## Conclusion

In conclusion, this study demonstrates that bone marrow stromal cells induce stromal activation with CAF-like features, engage IL-6 signaling and promote metabolic remodeling in MM cells that is associated with reduced BTZ sensitivity. By integrating metabolomics, stable isotope tracing and patient proteomic data, we identify IL-6 as an important contributor to this stromal-induced metabolic phenotype, recapitulating key aspects of the metabolic remodeling observed during co-culture. These findings highlight the intimate interplay between cytokine signaling and metabolism within the tumor niche and suggest that targeting IL-6-mediated metabolic adaptations may represent a promising strategy to overcome TME-mediated drug resistance. Collectively, our findings identify metabolic remodeling as an important component of stromal-mediated drug adaptation in MM and position IL-6 as an important contributor to this metabolic remodeling.

## Electronic supplementary material

Below is the link to the electronic supplementary material.

Supplementary Material 1

Supplementary Material 2

Supplementary Material 3

Supplementary Material 4

## Data Availability

The datasets used and/or analyzed during the current study are available from the corresponding author on reasonable request.

## Abbreviations

ADP: Adenosine Diphosphate
AMO1: Human MM cell line (specific cell line name)
ALDH1A1: Aldehyde Dehydrogenase 1 Family Member A1
APRIL: A Proliferation-Inducing Ligand
BMSC(s): Bone Marrow Stromal Cell(s)
BTZ: Bortezomib
CAF: Cancer-Associated Fibroblast
CC: Co-Culture
cDNA: Complementary DNA
Ct: Cycle threshold (qPCR measurement)
DMEM: Dulbecco’s Modified Eagle Medium
DPYD: Dihydropyrimidine Dehydrogenase
FAP: Fibroblast Activation Protein
FBS: Fetal Bovine Serum
GALE: UDP-Galactose-4-Epimerase
GAPDH: Glyceraldehyde 3-Phosphate Dehydrogenase
GLRX: Glutaredoxin
GSSG: Oxidized Glutathione
IC50: Half Maximal Inhibitory Concentration
IL: Interleukin
LC-MS: Liquid Chromatography-Mass Spectrometry
MC: Mono-Culture
MED1: Mediator Complex Subunit 1
MM: Multiple Myeloma
NAD(P)+/NAD(P)H: Nicotinamide Adenine Dinucleotide (Phosphate) oxidized/reduced forms
NF-κB: Nuclear Factor kappa-light-chain-enhancer of activated B cells
NCOA2: Nuclear Receptor Coactivator 2
OXPHOS: Oxidative Phosphorylation
PCA: Principal Component Analysis
PBS: Phosphate-Buffered Saline
PHGDH: 3-Phosphoglycerate Dehydrogenase
PPP: Pentose Phosphate Pathway
qPCR: Quantitative Polymerase Chain Reaction
RGE: Relative Gene Expression
RPL15/RPL19: Ribosomal Protein Subunit L15/L19
RPS10/RPS11/RPS27A: Ribosomal Protein Subunits S10/S11/S27A
SSP: Serine Synthesis Pathway
TGF-β/TGF-β1: Transforming Growth Factor-β
TNF-α: Tumor Necrosis Factor-α
TME: Tumor Microenvironment
TW(s): Transwell(s)
U-^13^C: Uniformly ^13^C-labeled
UMPS: Uridine Monophosphate Synthetase
α-SMA: α-Smooth Muscle Actin
