# Supplementary material for: IL-6-driven metabolic reprogramming of the multiple myeloma microenvironment
Source: Cancer Metab. 2026 Aug 6;14:35. doi: 10.1186/s40170-026-00451-4 (PMC13746571; doi:10.1186/s40170-026-00451-4)
Supplement: Supplementary file 1 [file 40170_2026_451_MOESM1_ESM.pdf]

IL-6-driven metabolic reprogramming of the multiple myeloma microenvironment

María Matamala Montoya[1,2], Silke Eleveld[1], Esther A. Zaal[1], Celia R. Berkers[1*]

[1]Division of Cell Biology, Metabolism & Cancer, Department of Biomolecular Health Sciences, Faculty of Veterinary Medicine, Utrecht University, Utrecht, the Netherlands

[2]Biomolecular Mass Spectrometry and Proteomics, Bijvoet Center for Biomolecular Research and Utrecht Institute of Pharmaceutical Sciences, Utrecht University, Utrecht, Netherlands

Supplemental Information

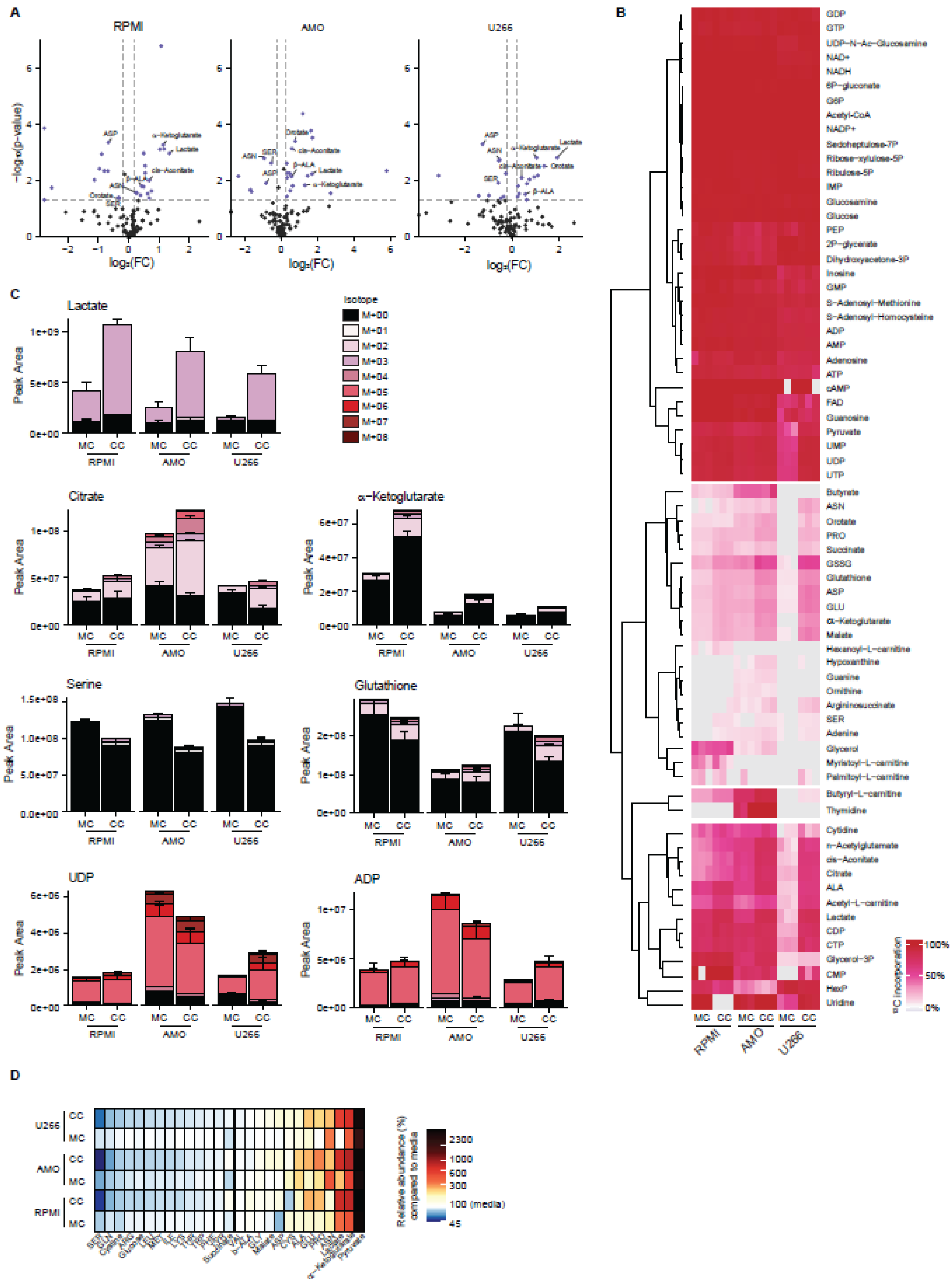

Supplementary Figure 1. Metabolic reprogramming in multiple myeloma cell lines under co-culture.

A) Volcano plots showing differential metabolite abundances in RPMI-8226, AMO1 or U266 cells comparing mono-culture (MC) *vs* co-culture (CC) after 48 h (n=3 replicates cultured in parallel wells). Mean metabolite levels were compared by Student's t-test and metabolites with p ≤ 0.05 are depicted in purple. Metabolites that were significantly altered in all cell lines are named. **B**) Hierarchically clustered heatmap of fractional ^{13}C-label incorporation into metabolites in RPMI-8226, AMO1, or U266 cells cultured under MC or CC conditions in the presence of [U-^{13}C]-glucose for 48 h (n=3 replicates cultured in parallel wells). Heatmap color scale represents the total labeled fraction (0-100%). **C**) Bar graphs showing isotopologue peak areas of lactate, TCA cycle intermediates (citrate, α-ketoglutarate), serine, glutathione and nucleotides (UDP and ADP) in RPMI-8226, AMO1, or U266 cells cultured under MC and CC conditions in the presence of [U-13C]-glucose for 48 h. Quantification of total peak area per metabolite. Data are presented as mean ± SD (n=3 replicates cultured in parallel wells). Different colors indicate different isotopologues. **D**) Heatmap of metabolites consumption (lower than 100%) or secretion (higher than 100%) in MC and CC of RPMI-8266, AMO1 or U266 cells compared to media, in the presence of [U-^{13}C]-glucose for 48 h (n=3 replicates cultured in parallel wells). Heatmap color scale represents relative metabolite abundance (45-2300%) compared to media (100%).

Supplemental Figure 2

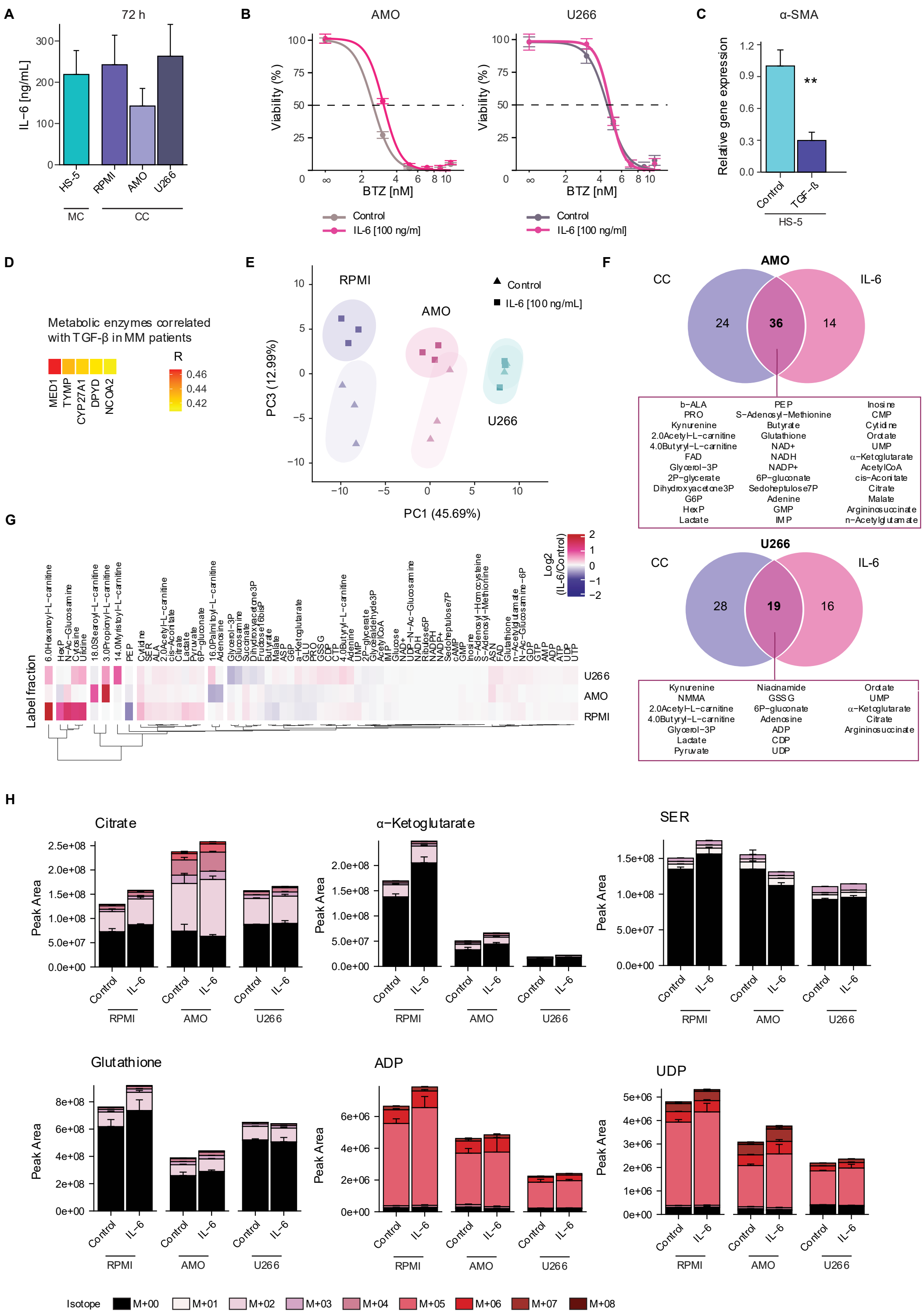

Supplementary Figure 2. IL-6 and TGF-β modulate drug response in multiple myeloma and stromal activation.

A) IL-6 levels measured by ELISA in conditioned media collected after 72 h from HS-5 mono-culture (MC), and co-culture (CC) with RPMI-8226, AMO1 and U266 cells. Data are presented as mean ± SD (n=3 replicates cultured in parallel wells). **B**) Dose-response curves of AMO1 and U266 cells treated with BTZ in the absence or presence of IL-6 (100 ng/mL). Cells were pre-treated with IL-6 for 24 h, followed by a by BTZ treatment at indicated concentrations for 48 h. Results represent % cell viability (mean ± SD, n=3 replicates cultured in parallel wells) compared to non-treated controls. **C**) Relative gene expression of FAP in HS-5 cells treated with 0 or 2.5 ng/mL TGF-β for 48 h. Data are represented as mean ± SD (n=3 replicates cultured in parallel wells), relative to untreated HS-5 controls. p-values were generated by Student's t test compared to 0 ng/mL TGF-β. ** $p \leq 0.01$. **D**) Heatmap showing metabolic proteins whose abundances correlate positively with TGF-β1 cytokine levels (Pearson's $R > 0.4$) in CD138+ plasma cells from MM patient in the MYELOMICS cohort. Color encodes the correlation coefficient. **E)** Principal component analysis (PCA) of total metabolite levels comparing treatment with 100 ng/mL IL-6 versus no IL-6 for 48 h (n=3 replicates cultured in parallel wells) in RPMI-8226, AMO1 en U266 cells. **F)** Venn diagram showing the up-regulated metabolites in AMO1 and U266 cells after co-culture with BMSCs or IL-6 stimulation, as compared to respective controls (n=3 replicates cultured in parallel wells). Data were obtained from 2 independent experiments. The overlapping region highlights metabolites that were consistently upregulated (fold change >0.05) under both conditions, which are listed below the diagram. **G**) Hierarchically clustered heatmap of fractional ^{13}C-label incorporation into metabolites in RPMI-8226, AMO1, or U266 cells cultured with 100 ng/mL IL-6 versus no IL-6 for 48 h in the presence of [U-^{13}C]-glucose for 48 h (n=3 replicates cultured in parallel wells). Heatmap color scale represents the total labeled fraction (0-100%). **H**) Bar graphs showing peak areas of all isotopologues of lactate in RPMI-8226, AMO1 and U266 cells treated without or with 100 ng/mL IL-6 in the presence of [U-^{13}C]-glucose for 48 h. Data are presented as mean ± SD (n=3 replicates cultured in parallel wells). Different colors indicate different isotopologues. **F**) Heatmap showing $\log_2$ fold changes (FC) in ^{13}C-label incorporation in IL-6 treatment *vs* control cells across RPMI-8226, AMO1, or U266 cells after culturing for 48 h with [U-^{13}C]-glucose (n = 3 replicates cultured in parallel wells). Fractional ^{13}C enrichment was calculated for each metabolite by summing peak intensities of all labeled isotopologues and dividing by the total peak intensity. Heatmap color scale represents $\log_2$ FC values of fractional ^{13}C incorporation in IL-6 *versus* control.

Supplementary tables

Supplementary Table 1. Primers sequences and annealing temperatures

Gene	Primer sequence	Ta (°C)
β-actin	Forward: GGA TGC AGA AGG AGA TCA CTG<br>Reverse: CGA TCC ACA CGG AGT ACT TG	60
RPL19	Forward: ATG AGT ATG CTC AGG CTT CAG<br>Reverse: GAT CAG CCC ATC TTT GAT GAG	61
α-SMA	Forward: ACT GAG CGT GGC TAT TCC TCC GTT<br>Reverse: GCA GTG GCC ATC TCA TTT TCA	60
FAP	Forward: TGA ACG AGT ATG TTT GCA GTG G<br>Reverse: GGT CTT TGG ACA ATC CCA TGT	58
FGF7	Forward: TCC TGC CAA CTT TGC TCT ACA<br>Reverse: CAG GGC TGG AAC AGT TCA CAT	59
IL-6	Forward: ACT CAC CTC TTC AGA ACG AAT TG<br>Reverse: CCA TCT TTG GAA GGT TCA GGT TG	58
IL1β	Forward: ATG ATG GCT TAT TAC AGT GGC AA<br>Reverse: GTC GGA GAT TCG TAG CTG GA	61
