# Supplementary material for: IL-6-driven metabolic reprogramming of the multiple myeloma microenvironment
Source: Cancer Metab. 2026 Aug 6;14:35. doi: 10.1186/s40170-026-00451-4 (PMC13746571; doi:10.1186/s40170-026-00451-4)

Supplemental Figure 2

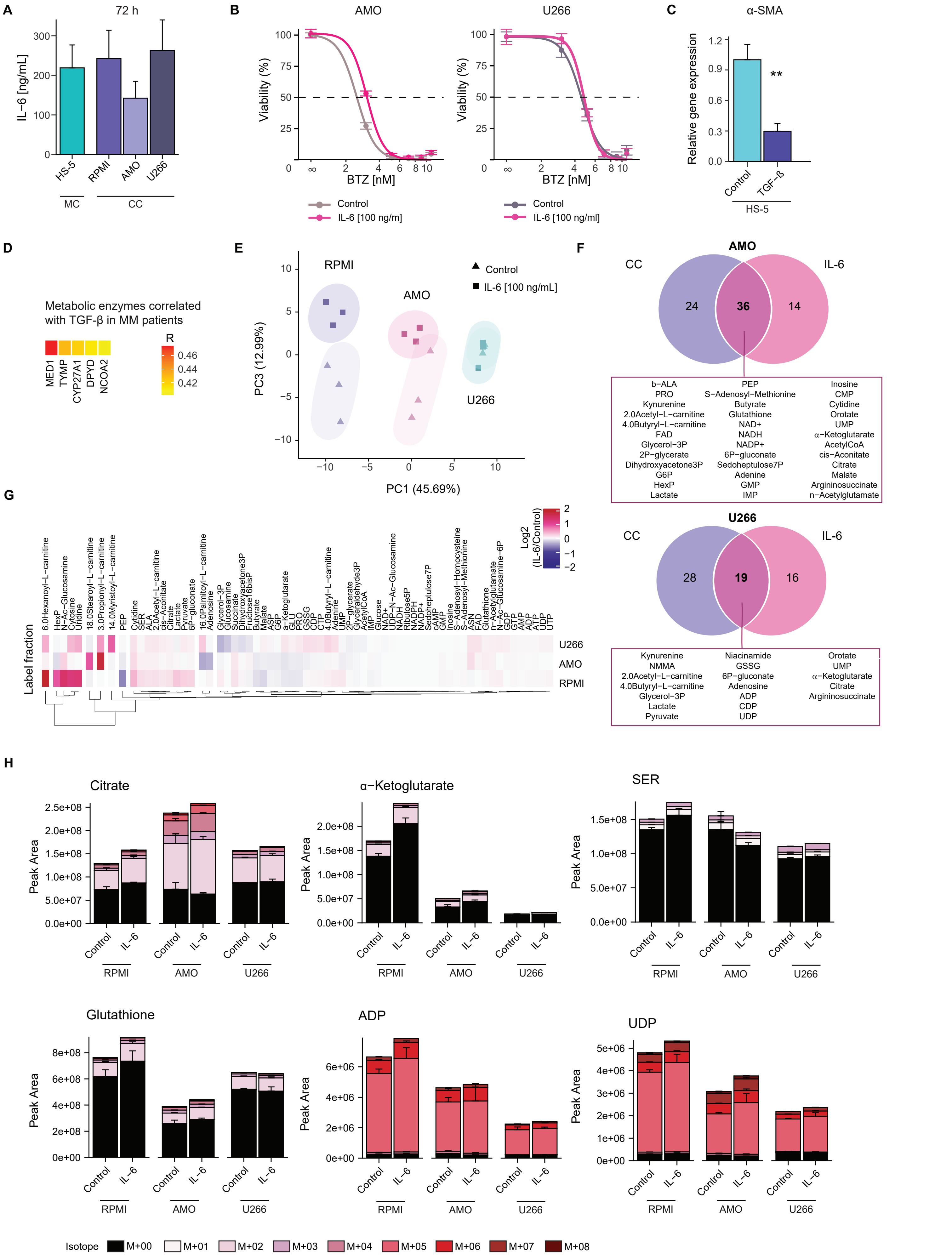

Supplement: Supplementary file 3 [file 40170_2026_451_MOESM3_ESM.pdf]
