# Supplementary material for: IL-6-driven metabolic reprogramming of the multiple myeloma microenvironment
Source: Cancer Metab. 2026 Aug 6;14:35. doi: 10.1186/s40170-026-00451-4 (PMC13746571; doi:10.1186/s40170-026-00451-4)
Supplement: Supplementary file 4 [file 40170_2026_451_MOESM4_ESM.pdf]

Supplementary tables

Supplementary Table 1. Primers sequences and annealing temperatures

Gene	Primer sequence	Ta (°C)
β-actin	Forward: GGA TGC AGA AGG AGA TCA CTG<br>Reverse: CGA TCC ACA CGG AGT ACT TG	60
RPL19	Forward: ATG AGT ATG CTC AGG CTT CAG<br>Reverse: GAT CAG CCC ATC TTT GAT GAG	61
α-SMA	Forward: ACT GAG CGT GGC TAT TCC TCC GTT<br>Reverse: GCA GTG GCC ATC TCA TTT TCA	60
FAP	Forward: TGA ACG AGT ATG TTT GCA GTG G<br>Reverse: GGT CTT TGG ACA ATC CCA TGT	58
FGF7	Forward: TCC TGC CAA CTT TGC TCT ACA<br>Reverse: CAG GGC TGG AAC AGT TCA CAT	59
IL-6	Forward: ACT CAC CTC TTC AGA ACG AAT TG<br>Reverse: CCA TCT TTG GAA GGT TCA GGT TG	58
IL1β	Forward: ATG ATG GCT TAT TAC AGT GGC AA<br>Reverse: GTC GGA GAT TCG TAG CTG GA	61
